# A generic method for evaluating crowding in the emergency department

**DOI:** 10.1186/s12873-016-0083-4

**Published:** 2016-06-14

**Authors:** Andreas Halgreen Eiset, Mogens Erlandsen, Anders Brøns Møllekær, Julie Mackenhauer, Hans Kirkegaard

**Affiliations:** Research Centre for Emergency Medicine, Aarhus University Hospital, Aarhus, Denmark; Department of Public Health, Section of Biostatistics, Aarhus University, Aarhus, Denmark

**Keywords:** Crowding, Emergency department, ED, Generic, Method, Model, Queue, Patient flow

## Abstract

**Background:**

Crowding in the emergency department (ED) has been studied intensively using complicated non-generic methods that may prove difficult to implement in a clinical setting. This study sought to develop a generic method to describe and analyse crowding from measurements readily available in the ED and to test the developed method empirically in a clinical setting.

**Methods:**

We conceptualised a model with ED patient flow divided into separate queues identified by timestamps for predetermined events. With temporal resolution of 30 min, queue lengths were computed as Q(t + 1) = Q(t) + A(t) – D(t), with A(t) = number of arrivals, D(t) = number of departures and t = time interval. Maximum queue lengths for each shift of each day were found and risks of crowding computed. All tests were performed using non-parametric methods. The method was applied in the ED of Aarhus University Hospital, Denmark utilising an open cohort design with prospectively collected data from a one-year observation period.

**Results:**

By employing the timestamps already assigned to the patients while in the ED, a generic queuing model can be computed from which crowding can be described and analysed in detail. Depending on availability of data, the model can be extended to include several queues increasing the level of information. When applying the method empirically, 41,693 patients were included. The studied ED had a high risk of bed occupancy rising above 100 % during day and evening shift, especially on weekdays. Further, a ‘carry over’ effect was shown between shifts and days.

**Conclusions:**

The presented method offers an easy and generic way to get detailed insight into the dynamics of crowding in an ED.

## Background

Crowding is a major health concern and an increasing problem for Emergency Departments (EDs) internationally [1–3]. Crowding is defined as a situation where treatment demands exceed available resources and is associated with increased mortality, increased treatment costs and over-all reduced quality of care [4–8].

Crowding can be conceptualised as a model of patient flow, which incorporates factors that influence on or are affected by crowding [9]. Any such model can be classified on a continuum from high generalisability with abstract information outputs (i.e. “level 1”) to highly specific with low generalisability (i.e. “level 4”) [10]. When developing a new model, researchers should acknowledge at which level they want their model to operate; balancing the need for application in various settings (level 1) against the need for a model drawing very specific conclusions (level 4). A simple generic method for evaluation of crowding that allows easy implementation into various EDs is in high demand [11–13].

The aim of this study was to develop a method that meets the criteria for an ideal universal measure for crowding being generalisable in measurement, definition and validity as described by Dr. Pines [14]. The proposed method was tested empirically in a clinical setting.

## Methods

### Study design and population

An emergency department (ED) has been defined as a hospital department accessible to patients with medical emergencies staffed by multidisciplinary clinical personnel ready to provide immediate stabilisation and care [15]. The processes that a patient must undergo from the time of arrival to departure characterise patient flow in the ED [9]. We defined a patient as being present in the ED from the time of electronic registration at the front desk (arrival) to the time of electronic registration of departure from the ED unit.

**Fig. 1:**
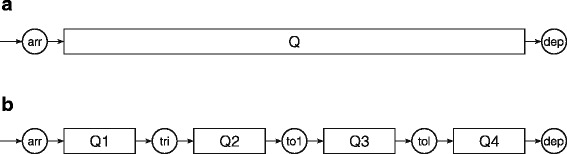
Conceptual models for the patient flow through the ED. **a** A simplified “black box” model of the patient flow to and from the ED depending only on time of arrival and departure. **b** By utilising surrogate-markers queues representing patients waiting on start of examination (Q1, waiting on triage), evaluation by a doctor (Q2, waiting on first TOKS), completion of examination/treatment in the ED (Q3, waiting on last TOKS) and leaving the ED (Q4) were proposed. All but a few patients in the ED would follow this flow in that exact order. Abbreviations: arr, arrivals; tri, triage; to1 first TOKS; tol, last TOKS; dep, departure, Q, queue

Since this study aimed at developing a generic method using readily available data to describe crowding we started by setting up a model of the ED depending only on the two most reliable timestamps: time of arrival and time of departure. This “black box” model has one queue representing ED census at a given time (Fig. 1a).

From the patient flow data, we created an aggregate data set by dividing the study period into intervals of 30 min and counting the number of arrivals A(t) and departures D(t) in each interval t (Table 1). From the number of arrivals and departures in each interval, the queue length at the beginning of the next interval could be calculated as: Q(t + 1) = Q(t) + A(t) – D(t), using Q(0) = 0 (the initial queue length).

**Table 1:** Variables obtained for each patient in each 30-min interval

Obtained data	Aggregated data set, 30-min intervals
Age	Mean age of patients present in the ED
Sex	
Time of arrival to ED	Number of patients waiting to be examined (Q1)
Time of triage	
	Number of patients waiting to be evaluated by a doctor (Q2)
Time of first TOKS	
	Number of patients waiting to have completed examination/treatment (Q3)
Time of last TOKS	
	Number of patients waiting to leave the ED (Q4)
Time of departure from ED	
Triage score	Number of patients in the ED triaged red
First TOKS score	
Last TOKS score	
Diagnosis	Number of trauma patients in the ED

Variables obtained from EHR for each patient registered in the ED in 2013 (left). From these variables an aggregated data set was compiled for 30-min. intervals calculating number of patients in each queue and number of trauma patients as well as patients triaged red (right). See also Fig. 1

*Abbrevations*: *ED* emergency department, *Q* queue, *TOKS* a Danish early warning system (see text for further explanation)

For every shift (day, evening and night) of each day in the study period the maximum queue (max queue) length was found. Days were defined as beginning by the day shift at 7 a.m. with every shift lasting eight hours, thus reflecting the clinical setting. To allow for steady state in the queuing system, all analysis of queues were made discarding the first 24-h, i.e. the first three shifts, of the study period.

To explore possible extensions of the model, thus increasing the level of information on throughput processes, we conceptualised an idealised patient flow by dividing the stay in the ED into four queues based on clinical relevance and structural challenges: A queue between arrival and start of initial assessment by an ED-nurse (Q1), a queue before evaluation by a doctor (Q2), a queue before completion of examination and treatment in the ED (Q3) and finally a queue arising for patients who are waiting to leave the ED (Q4). With few exceptions, e.g. trauma call patients, every patient presenting in the ED would go through these steps in that exact order. This is in line with the conceptual model proposed by Asplin and colleagues for ED throughput [9]. Since it was not possible to get verbatim timestamps for some of these factors (i.e. start of initial assessment, first evaluation by a doctor and completion of treatment), a number of surrogate-markers were decided upon: time of first triage, first re-assessment of vital signs “TOKS” value (a Danish early warning system based on systematic assessment of vital signs [16]) and last TOKS value. Figure 1b offers a graphical representation of this model.

In applying the method we used an open cohort design with prospectively collected data on every patient presenting to the ED from January 1^st^ to December 31^st^ 2013. The number of arrivals during the study period determined the sample size and patients were followed from ED admission to ED discharge as registered in the Electronic Health Records (EHR). EHR contains patient administrative data (e.g. age, gender, time of admission and discharge) and clinical patient data (e.g. triage score and diagnosis code). Utilising the “black-box” model for patient flow we computed the aggregate data set (*N* = 17,520) and max queue length for each shift (3) of each day (365, i.e. 3 * 365 values of max queues). Likewise, queue lengths for each of the four queues in the extended model of idealised patient flow in the ED were calculated for each day and shift (i.e. 4 * 3 * 365 values of max queues).

### Study site

We applied the method to the ED at Aarhus University Hospital, Denmark. The hospital had an uptake area of about 300,000 people and a census of approximately 70,000 annually divided on several units. The ED unit under study received about 40,000 patients in total divided on orthopaedic injuries, trauma patients and unstable medical patients. Other surgical patients and patients with medical conditions were also received although not exclusively in this unit. Some patient categories (including medical paediatric patients, patients with psychiatric emergencies and patients with heart related events) were not attended to in the studied ED but were transferred to specialised departments. Patients were either treated and discharged or admitted to the hospital. Physicians working in the ED had the right to admit patients to an appropriate inpatient bed, but in case of bed shortage on the wards, patients could be found boarding in the ED. Boarding time could not be defined since decision to admit were not registered. Unique department and location codes were used to record time of arrival and discharge from the studied ED unit. Hereinafter, the studied ED unit will be referred to simply as “the ED”.

The ED used Danish Emergency Process Triage (DEPT) for triaging patients on a five-point ordinal scale (1–5, 1, i.e. “red”, being the most acute) [17]. The capacity of the ED depends on available resources (i.e. number of nurses on duty according to the duty roster and number of available beds). Bed capacity in the ED was 19 with additionally two beds reserved for trauma patients (Table 2). On day and evening shifts, seven to eight nurses were on duty. On night shift four and five, nurses were on duty in weekdays and weekends respectively (Friday and Saturday night shifts were defined as weekend nights). When a trauma patient arrived at the ED, one to three nurses would be allocated to this patient thus lowering the nurse-capacity in the remaining ED. At times of special need one nurse could be transferred to the ED to increase the nurse-capacity though no formal staffing policy for this was in place.

**Table 2:** Characteristics of the emergency department and the patients

	Weekday (%)	Weekend (%)	Total (%)
Arrivals to the ED, 2013	29,715	11,978	41,693
Trauma	432 (1.45)	189 (1.58)	621 (1.49)
Triaged red	338 (1.14)	150 (1.25)	488 (1.17)
Female	13,848 (46.60)	5377 (44.89)	19,225 (46.11)
Age < 18 years old	7239 (24.36)	2682 (22.39)	9921 (23.36)
Age > 65 years old	5383 (18.12)	1866 (15.58)	7249 (17.39)
Length of stay ≤ 30 min	–	–	1120 (2.69)
Length of stay ≥ 5 h	–	–	2282 (5.47)
Number of beds ^a^	–	–	19
On-duty nurses	7–11.59 am: 7	7–10.59 am: 7	
	12–07.59 pm: 8	11 am–07.59 pm: 8	
	8–10.59 pm: 7	08–10.59 pm: 7	
	11 pm–6.59 am: 4	11 pm–6.59 am: 5	

Friday and Saturday nights were considered part of the weekend

^a^ The ED unit has two additional beds reserved for trauma call patients

### Statistical methods

Due to highly skewed distributions, we used non-parametric statistical methods to evaluate queue lengths: Kruskal-Wallis rank sum test to compare the distributions of the maximum daily queue length in each of the three shifts and between weekday and weekends and Spearman’s rank sum correlation test (Spearman’s rho) to evaluate correlations between maximum queue lengths in successive shifts. We applied Fisher’s index of dispersion to evaluate Poisson distribution of the data. Data management and statistics were done using R, version 3.1.2 (R Foundation for Statistical Computing, Vienna, Austria).

## Results

From the timestamps of arrivals and departures a simple method could be set up and risk of crowding and its predictors computed. It was possible to extend the model by separating the patient flow in the ED into clinically meaningful queues allowing for increased level of information. All codes necessary for the data management and analyses presented including an example data set are freely available (see section Availability of data and materials).

**Fig. 2:**
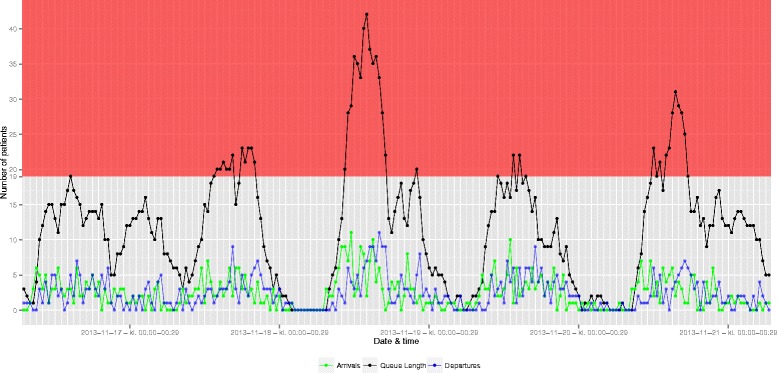
Time series plot offering insight on the queue dynamics. Arrivals (*green line*), departures (*blue line*) and the resulting queue (*black line*) summarised every 30 min (dots mark the beginning of an interval) for consecutive days. The red area marks 100 % bed occupancy or more. The maximum queue length registered was 42 on the 18th of November. No trauma patients arrived this day and the first patient triaged red were registered around 8.30 p.m. It does not seem to be a decrease in departures (i.e. due to prolongation of handling of patients in the queue) rather an increase in arrivals (i.e. external processes) that drives the queue length in this case

**Fig. 3:**
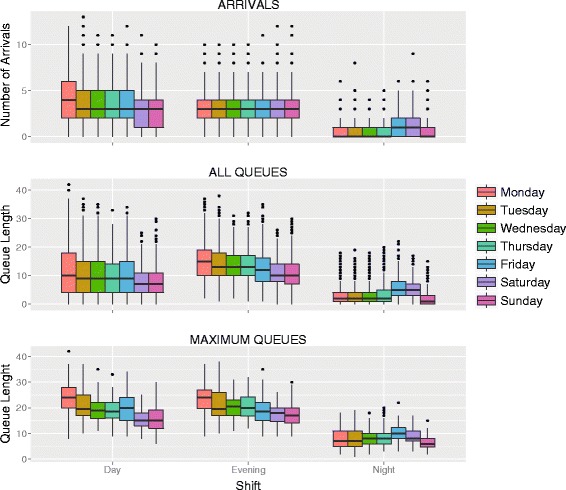
Boxplot of number of arrivals, queue lengths and maximum queue lengths per day in 2013. The distribution of arrivals and queue length were non-normal with over-dispersion. Queue lengths (“all” as well as “maximum”) and arrivals differed between shifts (Kruskal-Wallis: *p* < 0.0001). This was true as well between weekdays and weekends for maximum queues and arrivals, but for all queues an association was found (Kruskal-Wallis: *p* = 0.1309)

**Fig. 4:**
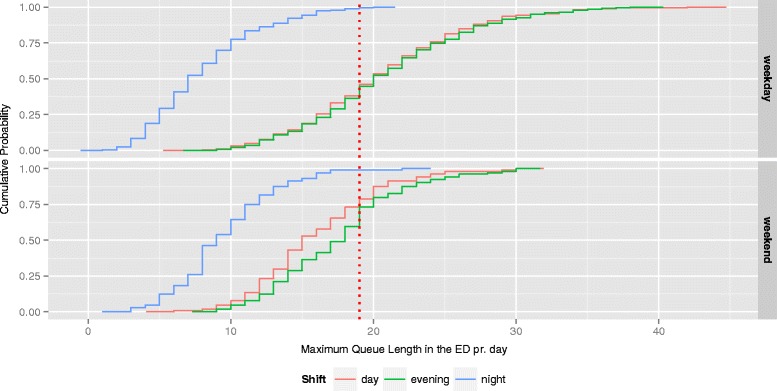
The empirical cumulative distribution function of max queues in the ED. The dotted red line indicates the point of 100 % bed occupancy (i.e. a queue length of 19). On weekdays there was a risk that 19 or more patients being present in the ED of 54 %, 56 % and < 1 % at some point during day, evening and night shift respectively. In weekends the risk was 21 %, 27 % and < 1 %

We tested the proposed method empirically on the “black box” model: A total of 41,693 arrivals were registered in the ED during the study period (Table 2). About 70 % of all arrivals happened on weekdays. Of all visits, 2.7 % (1120/41,693) spend 30 min or less in the ED and 5.5 % (2282/41,693) spend more than 5 h in the ED. Figure 2 offers detailed insights into the queue dynamics, illustrated by a queue of 42 patients, which arose on November 18th 2013. On this day the longest queue during the study period was observed, but any days of interest could have been chosen. The figure permits direct temporal observation of the queue length and its relation to the number of arrivals and departures indicating whether the queue length grows due to external or internal processes. If a fall in departures precedes prolongation of the queue length we expect internal processes (i.e. throughput) slowing down to be the main driver of the queue length. Conversely, a steep rise in arrivals (i.e. input) preceding a prolonged queue indicates external processes to be the driver of the queue length. It is always interplay between input, throughput and output, but in the example given in Fig. 2 external processes - i.e. the number of arrivals - appears to be the main driver of the queue length. The distributions of the number of arrivals and the queue lengths are shown in Fig. 3. These distributions could not be described by Poisson distributions since Fisher’s index of dispersion consistently showed over-dispersion, indicating negative binomial distributions would be a better fit. The distribution of max queues on weekdays vs. weekends and the three shifts differed significantly with Kruskall-Wallis: *p* < 0.0001 for both. On weekdays in day and evening shift the risk that the queue would grow to and above the maximum bed capacity at least once during the shift was more than 50 % (Fig. 4). On weekends this risk dropped to 21 and 27 % respectively. In night shifts the risk plummeted to around 1 % disregarding weekday/weekend. Seasonality (summer: April - September) was observed (Kruskall-Wallis: *p* = 0.0233), but only conveyed minor changes in risk of reaching a 100 % bed capacity when stratified for (e.g. the risk on weekday day shift were 54 % overall and 58 % and 50 % summer and winter respectively). The absolute number of times with crowding can be inferred from the percentages given in Fig. 4. Likewise, this frequency can be visualised with modifications to Fig. 2.

When restricting the analysis to include the observed max queue for each day and each shift, it was found that the median queue length was 20 patients with an interquartile range (IQR) of 8.00 patients on weekdays day and evening shifts, and 7 (IQR = 5.00) patients in the night (Table 3). On weekends the shift medians were 15 (IQR = 6.00), 18 (IQR = 6.00) and 9 (IQR = 4.25) patients respectively. This gives a median patient-to-nurse ratio of 2.9 in day shifts on weekdays as the maximum and 1.8 on night shifts as the minimum ratio detected.

**Table 3:** Characteristics of maximum queue lengths per day in 2013

		% of time with ≥19 patients	Min	Max	Median (IQR)	Median patient/nurse ratio
Day shift (7 am–02.59 pm)	Weekday	54 %	8	42	20 (8.00)	2.9 (2.5 when 8 nurses)
	Weekend	21 %	6	30	15 (6.00)	2.1 (1.9 when 8 nurses)
Evening shift (3 pm–10.59 pm)	Weekday	56 %	9	38	20 (8.00)	2.5 (2.9 when 7 nurses)
	Weekend	27 %	9	30	18 (6.00)	2.1 (2.4 when 7 nurses)
Night shift (11 pm–6.59 am)	Weekday	<1 %	1	20	7 (5.00)	1.8
	Weekend	<1 %	3	22	9 (4.25)	1.8

Characteristics of max queues and the median patient/nurse ratio computed from this. When observations were restricted to the time of each day and shift with the highest patient load there were considerable differences in the patient pr. nurse ratio. Friday and Saturday night were considered part of the weekend

*Abbreviation*: *IQR* interquartile range

**Fig. 5:**
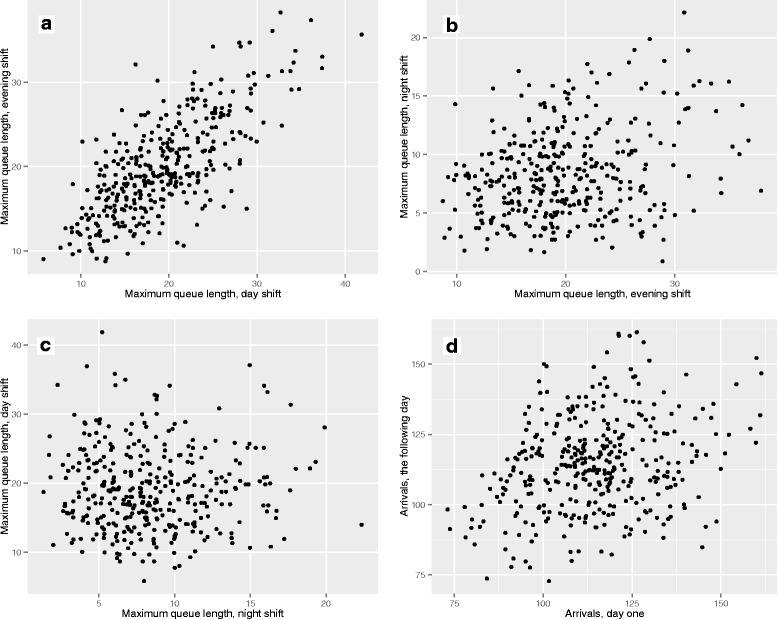
Scatter plot of relationship of max queue lengths between shifts and days following each other. The plots are jittered to avoid over plotting. Queue lengths correlated between shifts except for between night and day shift. Queue lengths also correlated between days (24-h) following each other. **a** Maximum queue on day and the following evening shift, Spearman’s rank correlation rho = 0.67, *p*-value < 2.2e-16. **b** Maximum queue on evening and the following night shift, Spearman’s rank correlation rho = 0.20, *p*-value = 0.0001. **c** Maximum queue on night and the following day shift, Spearman’s rank correlation rho = −0.02, *p*-value = 0.6496. **d** Arrivals on days following each other, Spearman’s rank correlation rho = 0.23, *p*-value = 7.498e-06

Max queue lengths in day shifts were found to correlate positively with max queue lengths the following evening shifts (*ρ* = 0.67, *p* < 0.0001) (Fig. 5). This relationship - a ‘carry over’ effect of queue length between shifts - also applied to evening and night shifts (*ρ* = 0.20, *p* < 0.0001), however, it did not apply to night and the following day shifts (*ρ* = −0.02, *p* = 0.65). Max queue lengths of one day correlated positively with max queue lengths the following day (*ρ* = 0.24, *p* < 0.0001).

**Fig. 6:**
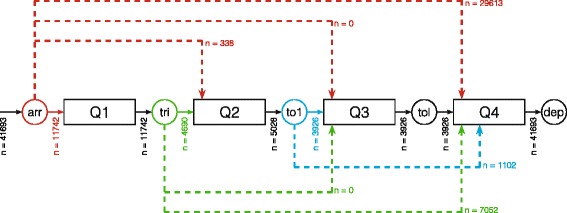
Examination of the empirical patient flow. Arrows indicates patient flow going from a timestamp to a queue. 41,693 patients arrived at the ED. Of these 11,742 (28.2 %) patients got a triage score registered, 338 (0.8 %) patients did not get a triage score but did get a first TOKS score, and 29,613 (71.0 %) patients had neither triage nor TOKS values registered. Of the 11,742 patients that did get a triage score 4690 also got a first TOKS score. Thus, a total of 5028 (12.1 %) patients had a registered value for first TOKS. Only 3926 (9.4 %) patients had a registered value for all the factors in the idealised model for the patient flow. It was therefore decided to do the further analysis on the “black box” model with only arrival and departure as factors, until better completeness of the data could be obtained. Abbreviations: n, number of patients; arr, arrivals; tri, triage; to1, first TOKS; tol, last TOKS; dep, departure; Q, queue

Unfortunately, in the explorative phase of the project it became clear that triage and TOKS data could not be retrieved for a large proportion of patients. For instance, all but arrival and departure registrations were missing in 71 % of the patients (Fig. 6). Thus, the above-presented calculations could not be repeated for the extended model. In an ED with data that supports this model each of the four queues could be analysed in a similar manner.

## Discussion

The main purpose of our study was to develop a generic method to describe and estimate crowding in an ED. By utilising only time of arrival and time of departure it was possible to get detailed insight into the dynamics of the queue and the resulting crowding in the ED. Since the method only relies on timestamps and patient census, it is the question at hand and data availability that sets the limit to the practical implementation of the method: A finer temporal resolution can be chosen, other time stamps can be used to define the queue of interest and several queues be combined to elaborate on the queues during patient flow (e.g. Fig. 1b). Likewise, the max queue can be related to one or more groups of staff in the ED and/or to patients of interest. We encourage fellow researchers to build on and modify the programming code for the method to suit the research question at hand.

The max queues represent the 30-min time period of each shift on each day with most patients in the ED. The interpretation is exemplified by the empirical cumulative distribution function (Fig. 4): It expresses the risk of *at least* one interval with a given queue length. Similarly, the positive correlation of max queues between shifts following each other means that, if the maximum queue during a shift was long, it was likely that a long queue would arise *at least* once during the following shift. When analysing crowding we are interested in what happens at times with prolonged queues. The observations with minor queues can be thought of as noise to the signal of interest. On the other hand if only observations with queue lengths above a certain threshold were chosen, variation would be lost and with it our ability to make inferences. To the knowledge of the authors, no other research has been done on maximum queues in the ED.

Crowding has been measured in a number of ways ranging from subjective assessment by ED staff [18] to objective measurements [19–24] and combinations of the two [12, 15]. To add to this, the threshold defining when crowding occurs is absolute in some studies [15, 19–21] using objective measurements for crowding and relative in others [24]. When crowding is defined subjectively the external generalisability should be questioned: What is perceived as crowding in one ED might be considered otherwise in another [25–27]. Objective definitions however, can be criticised for oversimplifying a complex interplay between numerous factors [9]. When the threshold for crowding is relative it is inevitable that crowding will occur a set percentage of the time, disregarding that the ED might never reach full bed capacity, experience the adverse effects of crowding etc. This limits possible research questions to concern effects of “above-normal” number of patients in the ED. Finally, dynamic measurement of crowding (i.e. several measurements during a patient’s stay in the ED) is preferable to a static measurement (i.e. a single measurement e.g. when arriving at the ED) [24]. Methods requiring highly detailed administrative data and/or specialised insight into statistical programming are seldom implemented in a clinical setting, are found to have low external validity and are not found to be superior to ED occupancy level in describing crowding [11, 13, 21, 28–30]. Likewise, if a method relies on specific assumptions about data, researchers must ensure that such assumptions are met before implementing the method. Failing to do so could result in deceptive conclusions. As an example, we found that arrivals did not follow a Poisson distribution, but rather a negative binominal distribution. This is in opposition to what is assumed in many studies on ED crowding [23, 24, 31] and could render such methods error-prone in our setting. Therefore, we sought to develop a novel method for quantifying crowding in an ED from simple and readily available parameters with occupancy level as a central measurement of crowding and a temporal resolution of 30-min.

One example of direct actions based on our proposed method is the ability to anticipate crowding: If the max queue in a day shift is long the ‘carry over’ effect mean that a long queue are likely to arise during the following evening shift. This will allow interventions to counter crowding before it arises. Such prediction tool could be implemented and evaluated using the Plan-Do-Study-Act tool [32].

Some limitations must be addressed. Arrival and departure time were recorded for every patient in the study and are generally considered very accurate, and possible errors in these timestamps were most likely random. If they were systematic, e.g. if at busy times the arrivals were systematically collected and registered with delay all at the same time, it could seriously bias the findings towards longer queues. Other indicators of flow were considered, amongst others time of diagnosis, time of first medicine prescription, time of ordering of x-ray and time of ordering blood tests. All of these were found to be particularly liable to “measurement” bias in that they would very often, and depending on the individual doctor, be registered later than actually effectuated, and were thus rejected. As with any standardised analysis model, this is an approximation of real life allowing a detailed overview of the system. Although very few assumptions are necessary with this method, complex organisational and psychological interactions between the factors are likely to play important roles in an ED; not least in times with crowding [33]. Such interactions have not been taken into account.

For the results of applying the method, it must be noted that EHR is a secondary data source primarily for clinical use. Since the data collection is not under the control of the researchers the quality of EHR data for research can be questioned [34]. On the other hand, the fact that data were collected as part of the clinical work without this research project in mind could be argued to lower the risk of introducing selection and/or information bias and thus strengthen the validity of the results. Further, the number of nurses on duty was derived from the duty roster and not the actual number of nurses meeting in each shift of each day. These numbers could differ e.g. due to illness. With a study period of one year we found it not to be feasible to allow for such variations but if investigating shorter periods of time this could be an option to strengthen the internal validity of the results. Finally, only the nurse capacity was included in the presented results leaving other valuable resources in the ED - such as doctors - unaccounted for. We encourage future research to focus on the possibility to extend the method to include several sub-queues (e.g. as proposed in Fig. 1b) and to explore the method in a multicentre trial.

In summary, building on the recommendations for dynamic measurement of crowding put forth by McCarthy et al. [24] we present a highly generic method relating ED capacity (beds and nurses) to dynamically measured ED census, setting no threshold for crowding. Our model is highly generalisable and easy to implement in diverse settings.

## Conclusion

We put forward a generic method for evaluating crowding in an ED from readily available data. This allows for detailed analysis of crowding: its impact and associations in any specified ED. It offers insight on the dynamics of crowding and allows for further investigation of predictors of crowding.

## Abbreviations

ED, emergency department; EHR, Electronic Health Records; IQR, interquartile range
